# Is Ataxia an Underestimated Symptom of Huntington's Disease?

**DOI:** 10.3389/fneur.2020.571843

**Published:** 2020-11-12

**Authors:** Gustavo L. Franklin, Carlos Henrique F. Camargo, Alex T. Meira, Giovana M. Pavanelli, Sibele S. Milano, Francisco B. Germiniani, Nayra S. C. Lima, Salmo Raskin, Orlando Graziani Povoas Barsottini, José Luiz Pedroso, Fernanda Aparecida Maggi, Vitor Tumas, Pedro Manzke de Carvalho, Ana Carolina de Oliveira, Bárbara Braga, Laura Cristina Souza, Rachel Paes Guimarães, Luiza Gonzaga Piovesana, Íscia Teresinha Lopes-Cendes, Paula Christina de Azevedo, Marcondes Cavalcante França, Alberto Rolim Muro Martinez, Hélio A. G. Teive

**Affiliations:** ^1^Movement Disorders Unit, Neurology Service, Internal Medicine Department, Hospital de clínicas, Federal University of Paraná, Curitiba, Brazil; ^2^Faculdade de Medicina, Universidade de Vila Velha, Espirito Santo, Brazil; ^3^Genetika – Centro de Aconselhamento e Laboratório de Genética, Curitiba, Brazil; ^4^Division of General Neurology and Ataxia Unit, Department of Neurology, Federal University of São Paulo – UNIFESP, São Paulo, Brazil; ^5^Movement Disorders and Behavioral Neurology Section, Ribeirão Preto Medical School, São Paulo University, Ribeirão Preto, Brazil; ^6^Departments of Neurology and Medical Genetics, School of Medical Sciences, State University of Campinas, Campinas, Brazil

**Keywords:** Huntington (disease), ataxia, cerebellum, chorea, polyglutamine (polyQ) diseases

## Abstract

**Background:** Huntington's disease (HD) is a progressive disorder characterized by motor, cognitive and psychiatric features. Cerebellar ataxia is classically considered as uncommon in HD clinical spectrum.

**Objective:** To determine the prevalence of cerebellar ataxia in patients with HD, both in the early and in the late stages of HD.

**Methods:** Seventy-two individuals considered eligible were assessed by two trained doctors, applying the Scale for Assessment and Rating of Ataxia (SARA) and Brief Ataxia Rating Scale (BARS) for ataxia, the Unified Huntington's Disease Rating Scale (UHDRS) and also, Barthel Index (BI), in order to evaluate functional capacity.

**Results:** Fifty-one patients (70.8%) presented with clinical ataxia at the time of examination (mean time of disease was 9.1 years). Six (8.33%) patients presented with cerebellar ataxia as first symptom. When stratified according to time of disease, a decline in the presence of chorea (*p* = 0.032) and an increase in cognitive deficit (*p* = 0.023) were observed in the patients as the disease progressed. The presence of ataxia was associated with longer duration of illness and severity of illness (UHDRS) (*p* < 0.0001), and shorter Barthel (less functionality) (*p* = 0.001).

**Conclusions:** Cerebellar involvement may play an important role in natural history of brain degeneration in HD. The presence of cerebellar ataxia in HD is relevant and it may occur even in early stages, and should be included as part of the motor features of the disease.

## Highlights

- Cerebellar ataxia is classically considered rare in Huntington's disease both at the beginning and throughout the course of the disease.
- Cerebellar involvement may contribute significantly to the understand of symptoms occurring in HD such as impaired fine motor skills, dysarthria, ataxia and postural instability, gait, and stance imbalance.
- Cerebellar ataxia may be underestimated, since the choreic symptoms are much more exuberant than other movement disorders, and it is possible much more prevalent than previously described in the scientific literature.

## Introduction

Huntington's disease (HD) is a neurodegenerative disease characterized by progressive motor, cognitive and psychiatric decline (1). HD has an autosomal dominant inheritance and is caused by CAG trinucleotide repeat expansion in the *HTT* gene (OMIM#613004) located on chromosome 4p16.3 (2). The age of onset is typically between 30 and 50 years, with a range of 2–85 years. The mean duration of the disease ranges from 17 to 20 years (3, 4).

Progressive motor dysfunction is a hallmark of HD, which usually starts subtly with slight involuntary movements, characteristically, chorea. With disease progression, further characteristic motor signs of HD emerge, such as dystonia, bradykinesia, and abnormal ocular movements. Notably, a small number of patients does not develop chorea, and may present with progressive rigidity and bradykinesia, constituting the clinical variant of Westphal, most commonly observed in juvenile forms of HD (4–6). Cerebellar ataxia is classically seen as an unusual symptom in HD, both at the beginning as throughout the course of the disease (5–9).

Ataxia represents a syndrome that is composed of numerous signs and symptoms that are characterized by the presence of gait ataxia, dystasia, dysmetria, dysdiadokokinesia, dysarthria, presence of pendular reflexes, kinetic tremors among others (10). Although some studies have demonstrated pathological changes in the cerebellum as Purkinje cells degeneration (11–13), clinical studies to evaluate cerebellar signs in HD are scarce.

It remains unclear whether cerebellar ataxia is underestimated in HD, since chorea is the predominant movement disorder, or if it is really a minor manifestation. Therefore, this study aimed to evaluate cerebellar ataxia based on neurological examination and clinical ataxia scales in different stages of patients with HD.

## Methods

### Selection of Patients

A multicentric cross-sectional study was performed, including patients with clinical and genetic diagnosis of HD. The study was conducted at the Neurology Service, Internal Medicine Department of the Federal University of Paraná in Curitiba, Federal University of São Paulo, University of Campinas and University of São Paulo (Ribeirão Preto). The study was approved by the ethics committee of each center. Informed and written consent was obtained from all patients.

One hundred and twenty-four patients with HD were enrolled in this study. Patients aged between 20 and 80 years, and all had genetic diagnosis of HD. Genetic diagnosis was applied according to the clinical criteria for HD, so that alleles with a CAG repeat length between 36 and 39 was defined as reduced penetrant (RP) and ≥40 as fully penetrant (FP) (14). Patients under 20 years of age were not included in order to avoid possible bias by including Juvenile Huntington's disease (JHD). Patients with advanced dementia, physical limitations, bedridden or asymptomatic were also excluded. From the initial 124 patients with HD, 72 patients were considered eligible for this study.

### Clinical Assessment

All 72 selected patients underwent clinical examination by two investigators. All investigators were neurologists trained to applied specific assessment tools. In order to decrease confusion bias, all patients were rated independently. The clinical protocol included: (1) Neurological examination with evaluation of cerebellar signs (dysmetria, dysdiadochokinesia, gait ataxia including tandem gait, or cerebellar tremor). (2) Application of Brazilian validated versions of Scale for the Assessment and Rating of Ataxia (SARA) (15, 16) and Brief Ataxia Rating Scale (BARS) (17, 18) to access ataxia severity. (3) Application of the Unified Huntington's Disease Rating Scale (UHDRS) (19, 20), for specific assessment of symptoms related to HD. (4) Application of Mini Mental State Examination (MMSE) and also the Barthel Index (BI) (21, 22), a functional assessment scale.

### Statistical Analysis

In order to describe the quantitative variables, mean, median, minimum, and maximum values and standard deviations were all considered, while frequencies and percentages were considered to describe the qualitative variables. The non-parametric Mann-Whitney test was used to compare two classifications of a qualitative variable regarding a quantitative variable. The association between two qualitative variables was evaluated considering the Fisher's exact test. The association between two quantitative variables was assessed by estimating Spearman's correlation coefficient. For multivariate analysis, the Linear Regression model (ANOVA) was used. The normality condition of the variables was assessed by the Jarque-Béra test. *P* < 0.05 was considered to be statistically significant.

## Results

From the 72 patients with HD evaluated, six (8.33%) patients presented with cerebellar ataxia as first symptom, manifesting as gait ataxia (Table 1). Clinical cerebellar ataxia was present in 51 patients (70.83%). Seventy patients had 3 points or more on SARA scale, representing 97.22% of the patients. Sixty-three (87.5%) patients had chorea; 65 (90.3%) patients had non-choreic movement disorders as dystonia, motor-tics; 62 (86.1%) patients had psychiatric features and 54 (75%) patients had cognitive function decline.

**Table 1:** Patients presented with ataxia.

**Variables**	**Patient 1**	**Patient 2**	**Patient 3**	**Patient 4**	**Patient 5**	**Patient 6**
Gender	F	F	M	M	M	M
Age	55	45	49	73	53	58
Age of onset	51	38	36	66	51	51
Duration of disease	4	7	13	7	2	7
Cerebellar findings	Gait ataxia, dysmetria, dysdiadococinesia	Gait ataxia. Mild dysmetria	Gait ataxia, dysmetria, dysdiadococinesia	Gait ataxia, dysmetria, dysarthria	Gait ataxia, dysmetria, dysarthria	Gait ataxia, dysmetria, dysarthria
Disproportionate cerebellar atrophy	Yes	No	No	No	No	No
Movement disorders	Chorea + dystonia	Bradikynesia	Chorea + dystonia	Chorea + dystonia	Chorea + dystonia	Chorea + dystonia
Cognitive and psychiatric findings	Anxiety	Dementia + behavioral	Dementia + behavioral	Dementia + behavioral	None	Dementia + behavioral
CAG	43	45	45	40	42	42
Pedigree	Father with chorea, dystonia	No family history	Father dead (with chorea)	Na	Na	Na
Inheritance	Paternal	Paternal	Paternal	Indefinite	Indefinite	Indefinite
SARA	9	7	9	12.5	10	2
BARS	8	7	6	11	8	3
UHDRS	28	41	61	35	31	12
BARTHEL	100	90	85	85	85	95

*BARS, Brief Ataxia Rating Scale; BI, Barthel Index; SARA, scale for the assessment and rating of ataxia; UHDRS, Unified Huntington Disease Rating Scale*.

**Figure 1:**
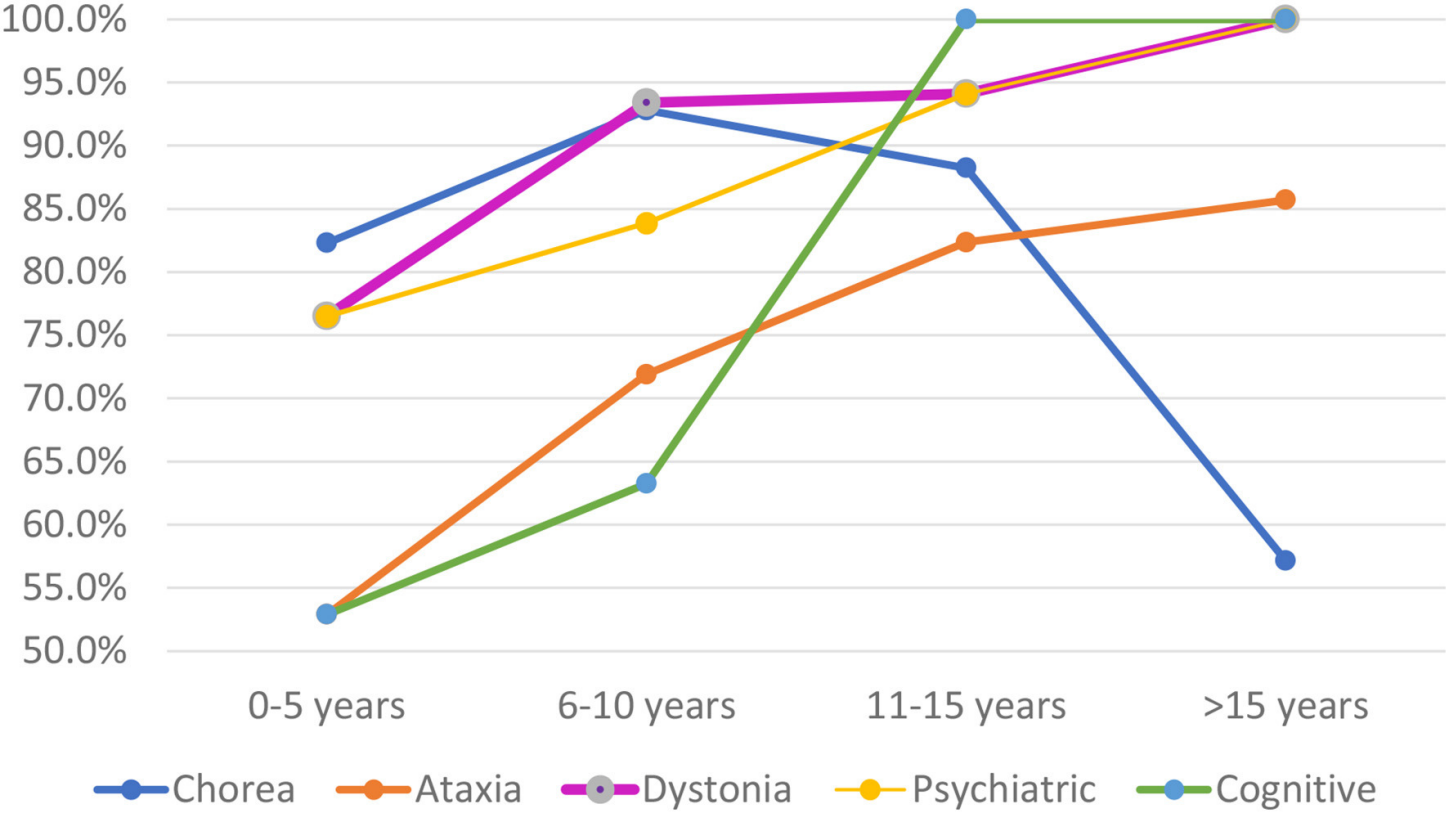
Evolutionary profile stratified according to time of disease.

When a correlation between the demographical data was performed according to time of disease, there was a decrease in the frequency of chorea (*p* = 0.032) and an increase in cognitive deficit (*p* = 0.023). However, as may be observed in Figure 1, chorea tends to be highly prevalent until 15 years of disease, after which it presents an inflection point and tends to be much less important, whereas ataxia keeps more present the longer the duration of the disease is, and the same occurring with dystonia, psychiatric, and cognitive disorders (Table 2).

**Table 2:** Clinical profile stratified according to time of disease.

	**0–5 years** **(*n* = 17)**	**6–10 years** **(*n* = 31)**	**11–15 years** **(*n* = 17)**	**>15 years** **(*n* = 7)**	p^*^
Chorea	14 (82.3%)	30 (92.8%)	15 (88.23%)	4 (57.14%)	**0.032**
Ataxia	9 (52.9%)	22 (71.9%)	14 (82.35%)	6 (85.71%)	0.214
Dystonia	13 (76.5%)	29 (93.4%)	16 (94.1%)	7 (100%)	0.276
Psychiatric disturbances	13 (76.5%)	26 (83.86%)	16 (94.1%)	7 (100%)	0.543
Cognitive function decline	9 (52.9%)	19 (63.25%)	17 (100%)	7 (100%)	**0.023**

* *Chi square test. Bold values indicates significance p < 0.05*.

The genetic expansion CAG ranged from 36 to 56 (mean 43.10 ± 3.91). Thirty-seven patients (51.3%) were female, and the mean disease duration was 9.14 ± 4.72 years. The disease duration ranged from 1.5 to 20 years. Paternal inheritance was more prevalent, which was present in 37.5% of the patients, 31.9% had maternal inheritance, 27.8% were indefinite and two patients (2.8%) had no family history (Table 3).

**Table 3:** Comparison of clinical aspects between groups with and without ataxia.

	**Total** **(*n* = 72)**	**Patients with ataxia** **(*n* = 51)**	**Patients without ataxia** **(*n* = 21)**	***p***
Gender	37F/35M	29F/22M	8F/13M	0.196^*^
Age	50.7(±11.8)	51.2 (±11.4)	49.7(±13.0)	0.515^**^
Age of onset	41.6(±11.2)	41.3 (±10.6)	42.45 (±12.82)	0.896^**^
Duration of disease	9.1 (±4.6)	9.9 (±4.47)	7.3 (±4.3)	0.016^**^
CAG	43.1 (±3.9)	43.3 (±4.16)	42.5 (±3.13)	0.667^**^
Inheritance paternal/maternal	27/23	21/14	6/9	0.228^*^
SARA	15.4 (±10.2)	18.5(±9.5)	7.95 (±7.7)	<0.0001^**^
BARS	13.1(±7.9)	15.5 (±7.4)	7.33 (±6.2)	<0.0001^**^
UHDRS	43.2 (±17.3)	48.6 (±15.3)	30.0 (±15.1)	<0.0001^**^
BARTHEL	79.3 (±23.7)	74.8 (±24.5)	90.2 (±17.6)	0.001^**^

* Fisher exact test.

** *Mann-Whitney's non-parametric test. Bold values indicates significance p < 0.05*.

When HD patients with ataxia (*n* = 51) were compared with HD patients without ataxia (*n* = 21), it was observed that patients with ataxia had significantly higher UHDRS indexes (*p* < 0.0001) and lower BI scores (*p* = 0.001; Table 3). The severity of ataxia (SARA) was associated with longer disease duration (rho = 0.43, *p* < 0.001), disease severity (UHDRS; rho = 0.75, *p* < 0.001), and decrease in functionality (Barthel; rho = −0.069, *p* < 0.001). CAG expansion and age at onset were not associated with ataxia (rho = *p* = 0.884 and rho = *p* = 0.532, respectively).

Breaking down the components of SARA, was found that the CAG expansion has no significant relationship with the SARA sub-items. Analyzing UHDRS, a positive correlation was observed with various components of SARA, such as gait, speech disturbance, sitting, fast alternating movements, in which the best correlation was found to be speech disturbance. When performing a multivariate analysis, in which the relationship of the CAG expansion and the UHDRS score was analyzed, relating to each SARA component, two models of significance were found, for the sub-items: speech disturbance and fast alternating movements (right and left), in which the greatest correlation found was also the model for speech disturbance.

## Discussion

This study showed that cerebellar ataxia is a common feature in patients with HD, and may occur early in HD, as six patients were observed to present with cerebellar ataxia as the initial manifestation. In this series, 70.8% of the patients with HD presented with variable degrees of cerebellar ataxia during the course of the disease, predominantly with gait ataxia. In this study, it was observed that chorea had a higher frequency during the first 6–10 years of disease, but it decreased with disease progression, becoming less prevalent after 10 years, in line with previous studies (3, 4). As expected, in our study a positive correlation between severity of the disease (UHDRS) and the cerebellar symptoms (SARA). Patients who had cerebellar ataxia as first symptom represented 8% of our sample, and did not have significant difference regarding CAG expansion, SARA or BARS. Of these, only one patient of our series had significant cerebellar atrophy observed on brain imaging.

In a cohort of 205 patients with HD, Squitieri et al. (23) described 2 patients who had limb and gait ataxia as the first clinical manifestation of HD, and 15 had atypical motor symptoms (non-choreic movements) at onset. Authors postulated that pathophysiological mechanisms in HD are multifocal, and not limited to impairment of the striatum. Also, Dong et al. (24) evaluated a sample which comprised 82 patients with HD, and identified 7 patients with an initial diagnosis mimicking spinocerebellar ataxia (SCA) due to the presence of ataxia as the first symptom. Other reports have also pointed out ataxia as an initial presentation of HD (25–27).

Considering clinical and genetic features, this study showed no correlation between CAG expansion and cerebellar signs. The presence of cerebellar ataxia was associated with longer disease duration and severity of HD (UHDRS). Squitieri et al. (23) also did not find statistical difference in the average CAG numbers and atypical onset motor symptoms and patients with typical ones.

Some pathological and neuroimaging data have demonstrated cerebellar involvement in HD. Singh-Bain et al. (7) performed a pathological study that observed cerebellar degeneration in patients with HD, characterized by loss of Purkinje cells in HD, that is more prominent with disease progression. Galvez et al. (28) evaluated 22 patients with HD through voxel-based morphometry (VBM) and concluded that levels of extrastriatal degeneration correlates with UHDRS scores. In the same study, the clinical motor changes in patients correlated with volume decrease of gray matter in the striatum, cerebellum, precuneus, insula, cingulate gyrus, middle occipital gyrus, and precentral gyrus. Ruocco et al. (29) observed a tendency to a positive correlation between cerebellar atrophy and duration of disease. They showed that there was a negative correlation of length of the CAG repeat with striatal degeneration, but its influence on extrastriatal atrophy, including the cerebellum, was not clear.

In this study, 97.22% of the patients had 3 points or more on SARA scale. Although the validation of SARA indicated that a score of 3 or more differentiates controls from patients with manifest ataxia (15, 30), we understand that other movement disorders may interfere with the interpretation of these data. This is the reason why we carry out a clinical evaluation. However, the finding of approximately all symptomatic patients with HD, presenting a quantified degree of ataxia, allows to enforce that cerebellar signs tend to appear early and be present in all stages of the disease, with the tendency to increase along the progression of the disease. On the other hand, 25% of the patients scored 10 points or higher on the SARA scale, despite having 40 points or less on the UHDRS scale, and several patients with very exuberant ataxia despite the fact that no important choreic/dystonic symptom were observed. These findings might suggest that, although a relationship between the severity of the disease (UHDRS) and the cerebellar symptoms (SARA and BARS) were observed, the presented relationship tends to move away from a linear fashion, which shows that probably the cerebellar degeneration does not occur proportionally to the already known striatal degeneration.

Although effort was made to minimize possible biases, it was understood that there are some limitations in this study that was taken into consideration to keep the quality of this work. Firstly, high stages of progression may show some degree of apraxia and it may input another confusion bias. Also, the presence of cognitive disorder is a characteristic of HD and an important contributor to disability. In the present study, patients with advanced dementia or who were not able to correctly perform the tests and maneuvers were all excluded.

In conclusion, this study demonstrated that cerebellar ataxia is common in patients with HD, and may be underestimated in clinical practice. Therefore, cerebellar ataxia should be included as part of the motor symptoms of the disease. Moreover, it is important to mention that ataxia may be the presenting symptom of patients with HD, eventually masquerading as spinocerebellar ataxia. And finally, this study shows that cerebellar involvement may play an important role in natural history of brain degeneration in HD.

## Data Availability Statement

The raw data supporting the conclusions of this article will be made available by the authors, without undue reservation.

## Ethics Statement

The studies involving human participants were reviewed and approved by Comitê de Ética em Pesquisa CEP-UFPR. The patients/participants provided their written informed consent to participate in this study. Written informed consent was obtained from the individual(s) for the publication of any potentially identifiable images or data included in this article.

## Author Contributions

GF and HT contributed to the initial development of the research, drafting, and review of the final manuscript. NL and AMe contributed to statistical analysis, and further development and completion. CC contributed to drafting, statistical analysis, and review of the final manuscript. SR contributed to the initial development of the research and genetic analysis of the patients. GF, FG, GP, AMe, and SM contributed to data and review. OB, JP, FM, VT, PC, AdO, BB, LS, RG, LP, ÍL-C, and PdA contributed to data, drafting, and review. GF was the lead author and takes responsibility for its overall content. All authors contributed to the development of the outline, revision of the manuscript, read and approved the final manuscript, and have ensured that this is the case.

## Conflict of Interest

The authors declare that the research was conducted in the absence of any commercial or financial relationships that could be construed as a potential conflict of interest.

## Abbreviations

BARS: Brief Ataxia Rating Scale
BI: Barthel Index
FP: fully penetrant
HD: Huntington's disease
JHD: Juvenile Huntington's disease
MRI: magnetic resonance imaging
RP: reduced penetrant
SARA: scale for the assessment and rating of ataxia
SCA: spinocerebellar ataxia
UHDRS: Unified Huntington Disease Rating Scale
VBM: voxel-based morphometry.
